# Health research system resilience: lesson learned from the COVID-19 crisis

**DOI:** 10.1186/s12961-020-00667-w

**Published:** 2020-12-18

**Authors:** Bahareh Yazdizadeh, Reza Majdzadeh, Ayat Ahmadi, Bita Mesgarpour

**Affiliations:** 1 Knowledge Utilization Research Center, Tehran University of Medical Sciences, Tehran, Iran; 2 Community-based Participatory Research Center, Knowledge Utilization Research Center, School of Public Health, Tehran University of Medical Sciences, Tehran, Iran; 3 National Institute for Medical Research Development (NIMAD), Tehran, Iran

**Keywords:** Coronavirus infections/prevention and control, Health policy, Pandemics/prevention and control, Policy making, Translational medical research

## Abstract

Producing evidence in epidemics is crucial to control the current epidemic and prevent its recurrence in the future. Data must be collected and analyzed rapidly to recognize the most efficient and feasible methods with proper timelines. However, there are many challenges a research system may encounter during a crisis. This article has presented lessons learned from the COVID-19 pandemic for health research system (HRS) to deal with current and future crises. Therefore, a HRS needs to produce and use evidence in such a situation. The components Knowledge Translation Self-Assessment Tool for Research Institutes (SATORI) framework was used to review the actions required and respond to the COVID-19 pandemic in a national HRS. This framework consists of four categories of defining the research question, conducting research, translating the research results, and promoting the use of evidence. The work is proposed actions in response to the COVID-19 crisis and improving a HRS's resilience. While COVID-19 has serious harm to the health and broader socio-economic consequences, this threat should be accounted for as an opportunity to make research systems more accountable and responsible in the timely production and utilization of knowledge. It is time to seriously think about how HRS can build a better back to be resilient to potential shock and prepare for unforeseen emerging conditions.

## Introduction

The COVID-19 epidemic has been a unique experience in highlighting the role of knowledge and research in solving a global challenge. Gaining control over the epidemic requires responses that are not possible except through knowledge generation, ranging from identifying the pathogenic agent's transmission routes to discovering the cure and prevention strategies, including the vaccine. More than ever, the human community is waiting for research solutions for an effective treatment or a vaccine for COVID-19. Therefore, the COVID-19 epidemic is an opportunity for research systems to improve and advocate the importance of their existence by being accountable in this crisis.

During this time, several articles have been published regarding the acceleration of the use of evidence in the control of the epidemic and its challenges and provided guidance based on the authors' previous research and experiences on how research systems must work.

Paul Glazio et al. have stated that the current research challenge is the quality of trial studies and the imbalance of their subjects, the quality of preprints, and many studies' repetitive nature [1]. One of the COVID-19 era changes is the pace of production of different ideas, depending on the time requirements, the implementation of research ideas, and disseminating results. In such a situation that the number of projects to granting bodies has increased, the conventional research committees' processes may not have enough capacity to deal with such proposals. Their supervision might get downgraded in some cases. At the same time, we have witnessed the most controversial retraction of articles during this period [2].

Increasing the number and variety of research products and research reports is not the only challenge that a health system research will encounter in crisis. The pressure from the public, social media, and politicians to respond to hot questions right away, alongside rumors and misinformation, puts a considerable burden on the health research system (HRS). This can easily deviate the real high priority research questions to hot research questions and waste resources [3]. Furthermore, in such time of pressure and tension, researchers are being asked frequently to answer burning questions like what best treatments are, when the vaccine would get released, and when the crisis will be over [4]. Therefore, it is not unexpected that some researchers become excited about their projects’ results and communicate them with the public even before their project gets completed. It would invoke lots of expectations from HRS, which needs to be managed as well. Dealing with such challenges requires modifying how we look at the production and use of knowledge, especially in resource-limited settings [5].

Iran was a country that, in late January, began imposing restrictions for the control of COVID-19. Public places were closed from April 23, 2020. During the Iranian New Year holidays, distancing measures were seriously followed. With the reduction of the infected cases, the preventive measures were relaxed under the banner of smart physical distancing from April. This increased the number of cases and subsequent surges. This led to a re-alert that if producing evidence and especially using it in decision-making does not sustain, it is likely to affect people's health.

This paper has a comprehensive look through emerging literature and what has been learned so far. Authors of the present have been active in the HRS for many years in Iran, where reported over 450,000 COVID-19 cases and 25,000 deaths since the first case notified on February 18, 2020, to the date of writing this paper (the beginning of October 2020) [6]. They have been working on formulating research questions and prioritizing them in association with the COVID-19 crisis in Iran and have contacted many research teams.

## Approach

The HRS's building blocks are ideal for a holistic overview of the system. This system's components are comprised of governance, finance, generation, and use of evidence and capacity building [7]. To clarify these components' status at the operational level, we used an institutional instrument to reveal the ground-level situation. The Knowledge Translation Self-Assessment Tool for Research Institutes (SATORI) framework [8] was used for this purpose. It has a holistic approach to 50 areas of assessing from choosing research questions to promote evidence processes. This tool was developed to set out all the needed dimensions, from defining the research question to conducting it, translating the research results and points that should be considered when promoting evidence. The SATORI has been developed based on knowledge utilization barriers and has suggested some interventions in each domain.

### Lesson learned

**Fig. 1:**
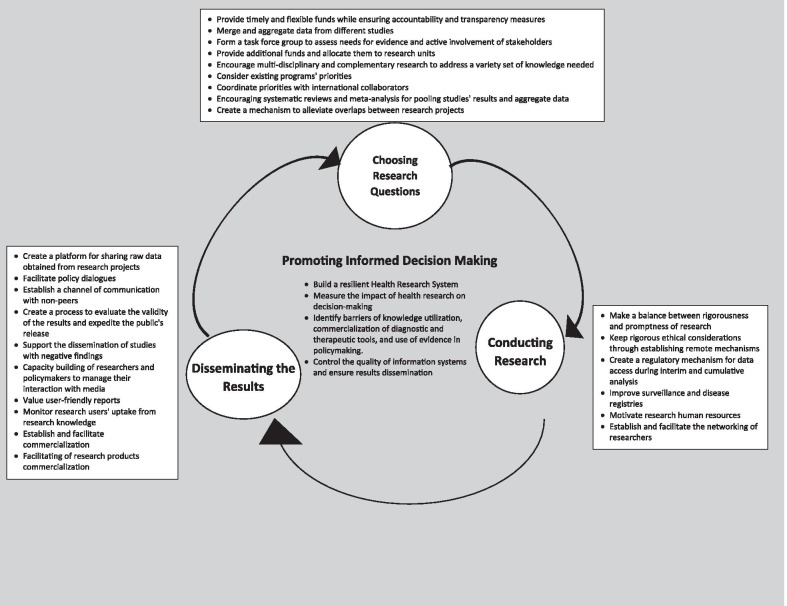
Interventions for building a resilient health research system

According to SATORI, topics are divided into four categories: How to choose research questions, how to conduct research, how to disseminate the results actively, and how to promote the use of evidence. Figure 1 summarizes the required actions based on SATORI tools.

#### Choosing research questions

One factor that increases the likelihood of using evidence in policymaking and decision-making is basing the research question's selection on the needs. During the COVID-19 crisis, various international organizations have carried out various activities to determine the knowledge needs at the global level [9]. Nevertheless, many research needs associated with local contexts may vary from country to country. Therefore, any country's research system should take measures to identify these needs and produce evidence that, in addition to helping to manage and control the crisis, can also contribute to preventing and controlling future crises. The suggested interventions are:

- Identify knowledge needs and determining real research priorities actively. It is recommended to form a task force group to assess evidence needs and actively involve stakeholders, including community and patients [10], health care providers, policymakers, and managers. Given the emergency condition, methods such as providing living evidence gap maps, media analysis, and knowledge brokers in the decision-making committees help have real-time research questions.
- Create a mechanism to alleviate overlaps between research projects is necessary. According to the World Health Organization recommendations, independent ethics committees should be linked to the central research granting committee to prevent approving repetitive projects [11].
- Merge and aggregate data to save time, increase reliability of results, and increase the research system's efficiency. To do this, it is necessary to actively coordinate various research teams to adhering the excellent research practice and follow a similar protocol, after the plans' approval, by keeping intellectual property transparent. It might be appropriate to mobilize public resources' research funding through national granting bodies as hubs for coordination and governing research.
- Encourage multi-disciplinary and complementary research to address a variety set of knowledge is needed. From one side, the system must understand the epidemic and the physiological, environmental, cultural, social, and economic factors affecting its onset and progression. On the other side, evidence on multifaceted interventions to prevent contagion, models for risk management, evidence on effectiveness and cost-effectiveness of interventions, and evaluating all the above at each stage the epidemic are needed [12].
- Give priority to pragmatic aspects of the programs, including monitoring and evaluation activities, documentation of learned lessons, and converting implicit knowledge to explicit knowledge.
- Coordinate priorities with international collaborators. Collaborators often have a broader scope and use cutting-edge technologies. GLOPID-R undertakes an initiative about the COVID-19 pandemic. It is an international network of major research funding organizations in the areas of virology, disease transmission and diagnosis, animal and environmental factors, epidemiology, clinical management, infection control, treatment effectiveness, vaccine production, ethics, social sciences and in collaboration with 400 experts and researchers have been identified [9].
- Encourage living systematic reviews and meta-analysis for pooling studies’ results, aggregate nationwide data would help to avoid research waste and get a comprehensive picture of the country's crisis. This option would answer some questions for the future epidemics and specifies the gap of evidence.
- Provide additional resources of funds and allocating them to research units if they can provide sufficient and qualified human resources and infrastructures. Guiding priority-based budget allocation to promote research on the needs in the country (urgent, short-term, and long-term) and forming an observatory group to monitor "already answered questions" and "ongoing research" would be helpful for efficient managing research priorities.
- Provide timely and flexible funding while ensuring accountability and transparency measures is crucial for mobilizing the health system to respond to emergencies.

#### Conducting research

The quality and quantity of performing research are highly dependent on researcher competency and motivations. Some of these motivations may not control HSR (such as journals' criteria for publishing research results). However, HSR can improve the national level of conducting research through regulation and set up motivations. Collected recommendations and suggestions to improve HSR ability in researching in crisis time are as below:

- Make a balance between rigorousness and promptness of research. In a crisis, conducting quick research but not at the cost of losing quality is a challenge. The WHO Solidarity trial is an example of timeliness of response while the quality of research met by recruiting several thousands of patients in different countries [13]. This ensures the quality of the evidence produced through active monitoring (quality control and quality assurance) and emphasizes the need for transparency in conducting project through registering protocols or data sharing and presenting the required documents.
- Enhance regulatory and ethical considerations through establishing remote mechanisms to evaluate ethics in proposals and tracing ongoing research through emphasizing transparency and sharing data. Moreover, modify these considerations, such as the requirement of Clinical Trial Authorization (CTA), for investigation of any new medical product or further indications of approved medicine. Giving priority to provide necessary protection for participants of trials on measures against the outbreak as well as other trials (e.g., cancer trials) should be considered in line with the ethical value of "benefiting others" [14].
- Create a regulatory mechanism to access the health data to do interim and cumulative analysis at the national level as well as for approved proposals: Data must be collected and analyzed based on the evidence required to make a decision (decision-driven data collection approach) by using the most efficient and feasible methods [15]. Given that every epidemic after a short time, the number of involved patients increased rapidly, cumulative data for assessing the heterogeneity of basic characteristics, diagnostic and therapeutic interventions, and quality of care would be necessary. However, the concern is always to respect the data owners' intellectual property, which should be managed through an ethical and professional process. On the other hand, the data related to clinical and basic sciences is directly related to public health decisions, so serious consideration should be given to the health system data's ownership to provide relevant evidence for policymaking without delay.
- Provide and improving the national system of disease surveillance and registry for patients' follow-up in the long term is required as a significant resource for continuity of data and using patients’ data more efficiently and timely.
- Apply incentives to keep researchers active, develop policies to prevent harmful competition, and support the potential resources to join international studies.
- Facilitate and establish a network of researchers doing similar researches while maintaining intellectual property makes their single activities more efficient.

#### Disseminating the results

Disseminating research results more often is performed through journal articles. To Improve and adopt the methods of communicating results, HSR can endeavor a variety of functions as below:

- Share the raw data obtained from the research, for example, creating a platform for the sharing raw data gathered from research with emphasizing on data sharing benefits for producing new evidence of the same sort of data.
- Establish a structure to facilitate policy dialogues sessions in studies aimed at improving policymaking.
- Identify a channel of communication with non-peers that considers all aspects of the intervention before dissemination.
- Create a process to evaluate the validity of the results and release them for the public faster than publication in scientific journals by considering researchers' intellectual property.
- Support dissemination of studies with negative findings to avoid duplication and malpractices.
- Prepare the guideline documents for researchers and policymakers to manage their interviews with the media and ask for avoiding publicity of their research's interim findings.
- Value user-friendly reports not less than publication in peer review journal considering quality appraisal and the level of produced evidence
- Monitor research users' experience with produced research knowledge.
- Establish and facilitate commercialization by activating the link between the research related to diagnostics, pharmaceuticals, and vaccines with the industrial sector. Following their previous studies on the time interval between discovering new drug interventions and their use in the population and its factors. Haney et al. designed a matrix and proposed interventions to reduce it and tailor those interventions to the conditions of COVID-19. This including increasing resources, working in parallel in different track matrix in a particular time, starting or working at risk (doing some actions in the next step despite safety and financial risk), and improving processes [16, 17].

#### Promoting the use of evidence

Performing programs and applying interventions need to be done after feasibility considerations. HSR can provide data and evidence on implementing a well-established evidence-based intervention and its effectiveness in the real situation*.* It is noteworthy that the interventions proposed in this section are the basis for strengthening other categories.

- Prepare HRS for emerging threats and be resilient to the unprecedented shocks in line with society, all governance, and the entire health system.
- Measure the impact of related research on decision-making at the national and sub-national levels and investigate why that is so and how it might be promoted
- Identify barriers to utilizing knowledge, commercialization of diagnostic and therapeutic tools, and use of evidence in policymaking.
- Control quality of information systems (such as health-related databases, registration and surveillance systems) and disseminating its results.

## Discussion

This article reviewed lessons learned for each segment of the processes, from choosing research questions to promoting the use of evidence processes during a crisis and subsequent changes needed for strengthening the HRS at the national level. Most of the interventions are related to the governance and generation, and use of evidence, other dimensions, including financing, well-elaborated within our analysis.

What is certain is that the COVID-19 epidemic will not be the last crisis we will experience in the coming years. We are already experiencing other kinds of emergencies, problems caused by emerging and re-emerging diseases or environmental disasters such as earthquakes and climate change. Therefore, this article emphasizes the lessons learned from the coronavirus pandemic for the research system.

Considering the urgency of research questions and new challenges that the system would face in crisis, research systems (in any condition) should learn from their data and retrieve further applicable information as soon as is available. It is worth noting that a research system's configuration in crisis highly depends on the research system's function in regular times. A research system's core functions are supposed to perform more effectively and rapidly rather than improve during the crisis [9]. Therefore, changing the structure and linkage between HRS components that have been built up over the years is not what we would like to recommend.

In general, to control crises, there are two approaches. The first approach is to predict it and prevent its occurrence or reduce its harmful effects when it is unavoidable. The second approach is to accept the unpredictable and even random nature of crises and deal with them by improving and strengthening the resilient systems [18]. Considering the second approach, we are currently responding to the COVID-19 crisis and extracting the lessons learned to improve the health system's resilience and the research system during future crises. Therefore, given the critical role of knowledge in the control and management of the crisis, countries' research system should provide the necessary evidence to control the upcoming crises and provide the infrastructure and facilities that ensure its resilience in crisis times. In other words, the interventions proposed by researchers in the field of knowledge translators must be implemented under normal circumstances so that they can be used in times of crisis as well. However, even in a well-established health system, it now can be reckoned that the conventional methods for performing the core functions of HSR might not be sufficient, and reinforcement of some characteristics may get indicated. Research system new demands, new audiences, and the urge to get answers from the public and policymakers may come up drastically. It shows that strengthening must be undertaken in all building blocks of the HRSs, including governance, financing, producing, and using evidence and capacity building, to address crisis challenges. These modification needs to be managed so that the core function of a research system does not collapse. Hence, new marginal constructions or reinforcing methods of performing regular operations seem necessary in crisis time. One of the system's most emphasized characteristics is how fast a system can learn and act according to its knowledge. In health services, such systems are called Rapid Learning Health Systems, and their attributes will encompass all functions and structures of a health system [19].

One of these facilities is creating a knowledge translation platform mentioned at the beginning of the article. Organizations such as McMaster Health Forum have also launched research knowledge infrastructures in recent years. During the COVID-19 epidemic crisis, they developed COVID-19 Evidence Network and Coronavirus (COVID-19)—Implement Cochrane resources and news to support Decision making (COVID-END) in a matter of weeks. Based on their experience on the Knowledge Translation platform, El-Jardali et al. have suggested new roles for the platform created during the COVID-19 epidemic. This includes engaging stakeholders in priority setting, synthesizing the best available evidence, contextualizing and disseminating actionable evidence to stakeholders, promoting trust and confronting misinformation, providing facilities for cross-sectoral dialogue, and monitoring and evaluating policy response [20].

The research systems of countries are different. To show these differences, we can refer to the building blocks of the countries' research systems. These blocks include stewardship, financing, creating and sustaining resources, producing, and using research [7]. The structures and processes of countries in each of these blocks are different. Therefore, countries must form structures and processes in each building block of research systems based on their context and infrastructure. In addition to being efficient under normal circumstances, they also have acceptable efficiency in times of crisis. In a crisis, policymakers must make tough decisions quickly, so predicting the data needed for the coming crisis in advance is crucial to Build Back Better [21]. In some countries, it may be necessary to change some processes in the building blocks in crisis times, but this change must be previously anticipated and feasible in crisis times. Therefore, countries must understand the most appropriate intervention in each block in critical situations. The COVID-19 pandemic is an opportunity to investigate the relationship between research system blocks and their impact in a regular and critical situation.

## Conclusion

Thus, national research systems must extract the lessons learned from the COVID-19 by accurately recording experiences, interventions and outcomes. At present, numerous measures have been undertaken in the country’s research system to face the COVID-19 crisis. It is worthwhile to evaluate them, extract the lessons learned, and consider establishing the necessary and possible structures and processes to deal with future crises. It means each HRS’s building blocks should be resilient to shocks and consider security as a goal for any system alongside producing and using evidence to improve health and its equity. Moreover, to be prepared for the next probable crisis, they must determine what changes need to be made in the country's infrastructure, management process, training the human resources, prerequisite evidence to be produced or updated, and informed decision making.

## Data Availability

Not applicable.

## Abbreviations

HRS: Health research system
SATORI: Knowledge Translation Self-Assessment Tool for Research Institutes
